# Beliefs and practices during pregnancy, post-partum and in the first days of an infant’s life in rural Cambodia

**DOI:** 10.1186/s12884-017-1305-9

**Published:** 2017-04-12

**Authors:** Claudia Turner, Sreymom Pol, Kamsan Suon, Leakhena Neou, Nicholas P. J. Day, Michael Parker, Patricia Kingori

**Affiliations:** 1 Cambodia Oxford Medical Research Unit, Siem Reap, Cambodia; 2 Faculty of Tropical Medicine, Mahidol University, Bangkok, Thailand; 3 Centre for Tropical Medicine and Global Health, Nuffield Department of Clinical Medicine, University of Oxford, Oxford, UK; 4 Angkor Hospital for Children, PO Box 50, Siem Reap, Cambodia; 5 Ethox Centre, Nuffield Department of Population Health, University of Oxford, Oxford, UK

**Keywords:** Neonatal, Pregnancy, Postpartum, Beliefs, Healthcare, Qualitative, Cambodia

## Abstract

**Background:**

The aim of this study was to record the beliefs, practices during pregnancy, post-partum and in the first few days of an infant’s life, held by a cross section of the community in rural Cambodia to determine beneficial community interventions to improve early neonatal health.

**Methods:**

Qualitative study design with data generated from semi structured interviews (SSI) and focus group discussions (FGD). Data were analysed by thematic content analysis, with an a priori coding structure developed using available relevant literature. Further reading of the transcripts permitted additional coding to be performed in vivo.

This study was conducted in two locations, firstly the Angkor Hospital for Children and secondarily in five villages in Sotnikum, Siem Reap Province, Cambodia.

**Results:**

A total of 20 participants underwent a SSIs (15 in hospital and five in the community) and six (three in hospital and three in the community; a total of 58 participants) FGDs were conducted. Harmful practices that occurred in the past (for example: discarding colostrum and putting mud on the umbilical stump) were not described as being practiced. Village elders did not enforce traditional views. Parents could describe signs of illness and felt responsible to seek care for their child even if other family members disagreed, however participants were unaware of the signs or danger of neonatal jaundice. Cost of transportation was the major barrier to healthcare that was identified.

**Conclusions:**

In the population examined, traditional practices in late pregnancy and the post-partum period were no longer commonly performed. However, jaundice, a potentially serious neonatal condition, was not recognised. Community neonatal interventions should be tailored to the populations existing practice and knowledge.

**Electronic supplementary material:**

The online version of this article (doi:10.1186/s12884-017-1305-9) contains supplementary material, which is available to authorized users.

## Background

Globally the proportion of childhood deaths that occur in the neonatal period is increasing [1]. Since 1990 there has been a 47% reduction in deaths in children less than five years of age [2]. However, this rate of reduction has not been seen in infants four weeks of age or younger. It is estimated that 2.9 million neonates die each year, with one million of these occurring on the first day of life [3]. Globally neonatal mortality now makes up 44% of all deaths in children younger than five years [3]. The reasons why the fall in neonatal mortality has not mirrored that seen in childhood mortality are complex. One proposed explanation is that interventions that have been successfully employed to reduce childhood death do not reach the community, where most neonates die [4, 5]. Another perceived barrier to providing neonatal care, particularly in remote areas, is the misconception that neonatal care is difficult and expensive [6]. During the pregnancy and the postpartum period, perhaps more than at any other time, there are deep rooted health practices and beliefs [7, 8]. Some of these practices are potentially harmful to the neonate and should be addressed in the communities where they occur.

Cambodia has one of the highest neonatal mortality rates in Southeast Asia. In addition, due to the lasting effects of civil war, there is a lack of trained healthcare providers, particularly in rural areas. The neonatal mortality rate is three times higher in rural areas than in urban areas [9]. In addition, there are only two functioning neonatal care units in the country and neonatal medicine is not nationally recognised as a speciality.

Understanding community practice and beliefs is a vital step in improving early neonatal outcomes as identifying knowledge gaps and harmful behaviour would allow community based programmes to be tailored to need. There is a paucity of data regarding practices and beliefs during the late stages of pregnancy and in the post-partum period in Southeast Asia [10]. Geographically representative data is important to be able to improve health care provision in the early post-partum period.

## Methods

This qualitative study was conducted in two parts. The first was based at the Angkor Hospital for Children (AHC), Siem Reap, Cambodia [11]. Semi structured interviews (SSIs) were conducted with mothers to examine maternal beliefs and FGD were conducted with other groups to enrich this data and obtain a general view of the population’s tradition beliefs around the time of childbirth.

All SSIs and FGD were conducted in Khmer by two trained Khmer field staff. For both the SSI and FGDs one of staff conducted the SSI or the FGD and one member of staff took notes (topic guides for the SSI and FGD can be seen in Additional files 1 and 2). Both sat together afterward and completed a contact summary sheet detailing nonverbal communication that occurred and significant points arising.

Fifteen SSIs with mothers from rural areas whose babies had been admitted to the neonatal unit were conducted over a three-month period. Questions concerning traditional practices, current practices and explanations of problems were asked around four areas: birth, the newborn period, neonatal illness and healthcare seeking were discussed. Examples of questions included:

“Do you know of any problems that can happen to a woman during labour and childbirth?”

“Are there any special things that must be done after a baby is born?”

“How do you know a newborn baby is healthy?”

“Whose responsibility is it to decide whether to take a sick baby for help?”

Mothers were selected using convenience sampling and had babies, who were medically stable but admitted to the neonatal unit. The study was explained to them and written consent obtained. Also at AHC, focus group discussions (FGD) were conducted with three separate groups: grandmothers, fathers and healthcare workers. Fathers and grandmothers who participated in the FGD were also chosen by convenience sampling: they all had children or grandchildren admitted to AHC.

**Fig. 1:**
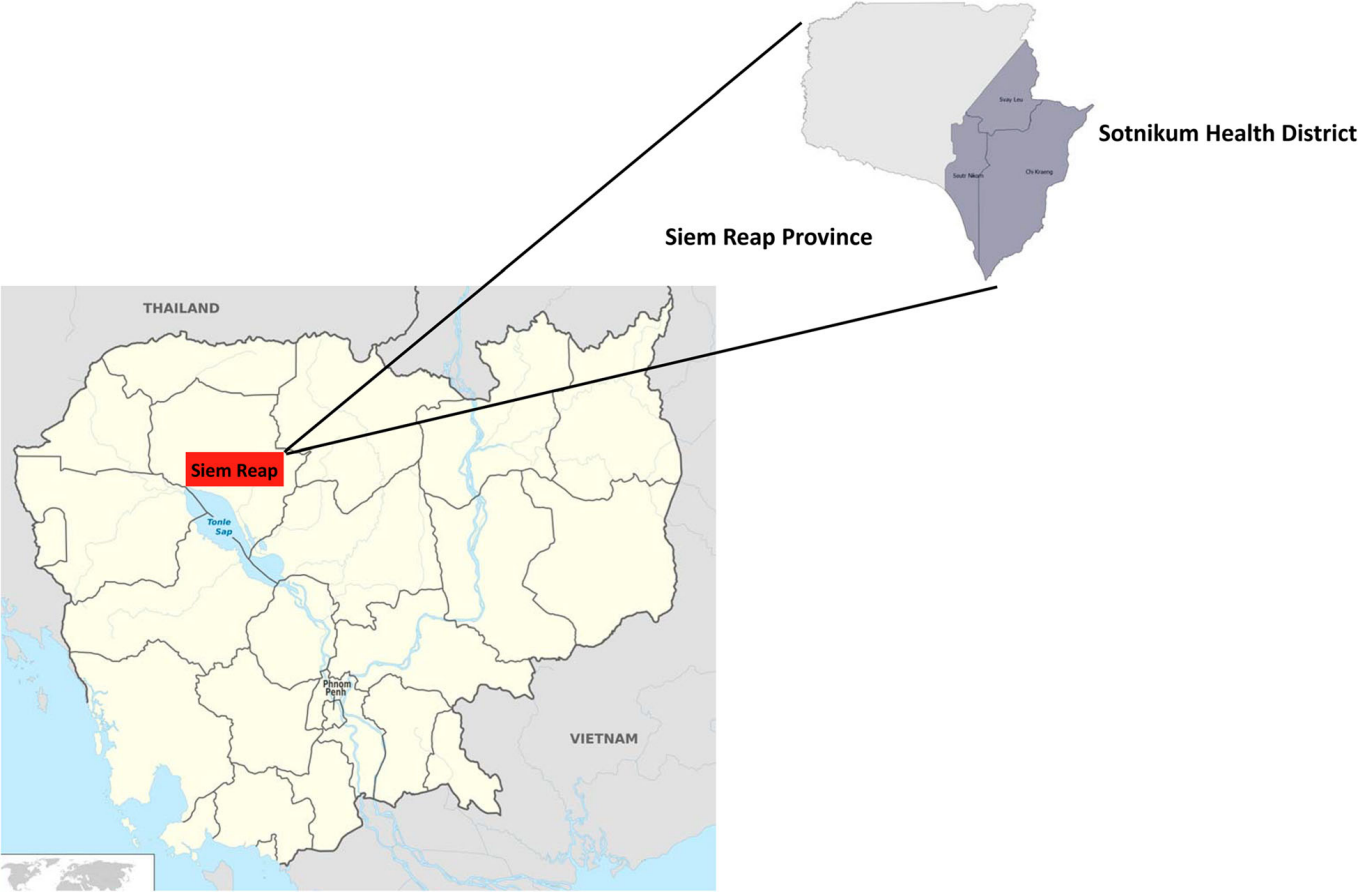
Map of Cambodia showing Siem Reap Province with Sotnikum Health district highlighted in grey (map adapted from Wikimedia commons https://commons.wikimedia.org/wiki/File:Cambodia_location_map.svg)

Following analysis of the hospital-based SSI and FGD the topic guides were amended to ensure capture of the emergent themes. A further five SSI were conducted with mothers of infants and three FGDs with community healthcare workers and village elders. The SSIs and FGDs were conducted in Sotnikum, a rural district located in Siem Reap Province, one of the poorest provinces in Cambodia (Fig. 1). Participants were chosen from rural areas using convenience sampling. During the study period a neonatal mortality village survey was being conducted by the study team, during the course of this survey villagers were invited to participate.

### Data management and analysis

All SSI and FGDs were voice recorded, translated (for meaning) and transcribed into English: the interviewer checked the transcripts against the recording to ensure meaning had been captured. The principal investigator (PI) then read the transcripts, checking the English was correct. Ambiguous or unclear language was discussed by the interviewer and PI until a consensus was reached. All data was imported into N-Vivo 10 (QSR International, Cambridge, MA). Coding was performed using a framework/thematic analysis by all members of the study team. After translation the Khmer field workers and PI re-examined the transcripts, using the observational notes and voice recordings to inform the coding structure and to ensure confirmability and dependability. The analytic approach taken was informed by the research question: to explore the beliefs and practices during pregnancy, post-partum and in the first days of an infant’s life in rural Cambodia.

Prior to the SSIs or FGDs a short socioeconomic questionnaire was completed by Khmer field workers who recorded participants verbal responses. These data wereentered into an Access 2003 database (Microsoft) and systematically checked for errors. These data were analysed using Stata/IC 12.1 (StataCorp, College Station, Tx, USA). Continuous variables were described by the median and range.

### Ethics statement

Ethical approval was obtained from the Cambodian National Ethic Committee for Health Research (0244 NECHR), the Oxford Tropical Research Ethics Committee (535–14) and the AHC Institutional Review Board (676/14).

## Results

**Fig. 2:**
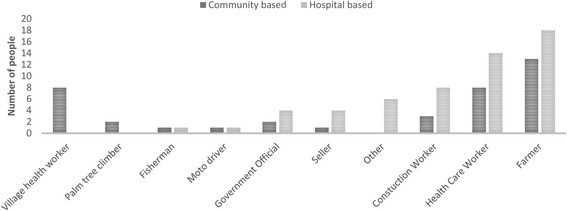
Graph showing occupation by hospital and community groups (a seller is a small market trader, usually of household goods, a palm tree climber is someone who scales palm trees to harvest its fruits or sap which is refined into sugar)

A total of 20 SSIs (15 in hospital and five in the community) and six (three in hospital and three in the community: total 58 participants) FGDs were conducted. Socioeconomic characteristics are shown in Table 1 and Fig. 2.

**Table 1:** Socioeconomic factors of participants in hospital and community based SSIs and FGDs

	Hospital based (% of all study participants)	Community based (%of all study participants)
Participants in the study	49 (62.8)	29 (37.2)
Women, n (%)	32 (65.3)	18 (62.1)
Mothers, n (%)	18 (36.7)	14 (48.3)
Fathers, n (%)	14 (28.6)	5 (17.2)
Grandmothers, n (%)	9 (18.4)	3 (10.3)
Grandfathers, n (%)	0	3 (10.3)
Age in years, median (range)	31 (18–69)	38 (20–59)
Number of own children, mean (range)	2 (1–9)	4 (1–10)
Participants who had had a still birth, n (%)	1 (2.0)	5 (17.2)
Participants who had had a child die, n (%)	7 (14.3)	9 (31.0)
Education		
Primary not completed, n (%)	12 (24.5)	7 (24.1)
Primary completed, n (%)	1 (2.0)	4 (13.8)
Lower secondary school, n (%)	9 (18.4)	5 (17.24)
Secondary school diploma, n (%)	4 (8.16)	4 (13.8)
Beyond secondary school, n (%)	16 (32.7)	0
Housing		
Permanent structure, n (%)	39 (79.6)	24 (82.8)
Temporary structure, n (%)	10 (20.4)	4 (13.8)
Number of rooms, median (range)	1 (1–4)	1 (1–5)
Number of occupants, median (range)	5 (1–9)	5 (1–11)
Toilet in the house, n (%)	32 (65.3)	16 (55.2)
Mains electricity, n (%)	32 (65.3)	11 (37.9)
Piped water, n (%)	12 (24.5)	7 (24.1)
Fridge, n (%)	8 (16.3)	0
Roof		
Thatched, n (%)	4 (8.2)	4 (13.8)
Metal sheet, n (%)	3 (6.1)	2 (6.9)
Tiles, n (%)	14 (28.6)	10 (34.5)
Zinc, n (%)	22 (44.9)	13 (44.8)
Mode of transportation		
Bicycle, n (%)	21 (42.9)	14 (48.3)
Motorcycle, n (%)	30 (61.2)	18 (62.1)
Car, n (%)	1 (2.0)	4 (13.8)
None, n (%)	12 (24.5)	6 (20.69)
Animals owned		
Water buffalo, n (%)	1 (2.0)	1 (3.4)
Chickens/duck, n (%)	23 (46.9)	20 (69.0)
Cow, n (%)	7 (14.3)	10 (34.5)
Pig, n (%)	3 (6.1)	4 (13.8)
Family Monthly income		
< 60 USD, n (%)	11 (22.5)	9 (31.0)
60–120USD, n (%)	10 (20.4)	12 (41.4)
120–240 USD, n (%)	12 (24.5)	6 (20.7)
> 240 USD, n (%)	14 (28.6)	2 (6.9)

### Mothers

Traditional practices during pregnancy, such as food restriction, were not universally practiced. However when they were they typically focused on the mother. When interviewees were asked why these practices were for mothers and not babies a common response was that the baby was not unwell but that the mother was in danger of being ill, therefore the mother needed assistance as the baby was reliant on the mother.

*“When deliver successfully, baby don’t get danger. Only for the mother it can be dangerous.”* [Grandmother]

Traditional medicine was commonly described as being used at two time points, one month before birth, to help the baby to be born more easily and postpartum to increase breast milk production and prevent Tos. Tos has no translation in English. It is a physical and or psychological condition that postpartum women can suffer from if they eat the wrong food, partake of wrong actions, thoughts or emotions or injure themselves. The described symptoms of tos range from collapse, seizure, locked jaw, “madness” to abdominal pain and diarrhoea. Use of different plants were described, most of which were brewed in hot water to make a tea.

None of the women interviewed described food restriction in pregnancy. In fact, weakness and difficult labour was blamed on not eating enough during pregnancy and women described eating more so they would be strong to deliver their baby.

*“We have to eat enough food during pregnancy to make the baby strong. We have to eat nutritious food” [Mother]*

### Birth and post-partum

Where mothers chose to have their babies was discussed in the FGDs and SSIs. The majority of women reported that they felt that a baby should be delivered in a health facility. A common reason given for this was the Cambodian governmental policy to stop women delivering at home with a traditional midwife.

*“yes, a traditional midwife can be arrested at any time if they deliver the baby. If they still insist to deliver with a traditional midwife and if health centre staff know about it the traditional midwife will be sued. So, when our baby gets sick and bring to the health centre, the health centre staff will not take any responsible if there is a problem.” [Father]*

A few mothers mentioned that they needed to go to the health centre four times during pregnancy to get a check-up.

Many of the interviewees and FGD members and said that the umbilical cord was for the baby to breathe through and to get food by sucking on it. There was no description of using anything other than scissors to cut the cord and none of the mothers had used traditional medicine on the cord although one mother did describe an old practice of using a paste made from a wasp’s nest on a child’s umbilical cord stump. Here a mother recalls instructions she has been given by the midwife and how this conflicts with traditional beliefs:

*“The midwife asks us not to put anything on the umbilical cord because it might cause problems to the baby. But traditional midwife tell me to put some soil from the roof [wasp nest] on the umbilical stump to heal it”* [Mother]

All of the mothers said that a baby should be fed straight after birth and that colostrum should not be discarded, although a few did mention that this had been the practice in the past.

*“After birth we start breast feeding the baby. Even though mother’s breast not yet produce milk. We allow baby to suck on the breast to stimulate in order to quickly have milk”* [Healthcare worker]

### Traditional beliefs

Not all of the mothers interviewed held traditional beliefs. Some mothers did not take traditional medicine or conduct traditional ceremonies and professed a greater confidence in modern medicine rather than traditional medicine.

Some women described having modern medicine during birth and then using traditional medicine when that had finished. Traditional medicine consisted of different plant leaves, bark and roots. There were also a number of mothers who said that they had used traditional medicine in an earlier pregnancy but were now only using modern medicine. One explanation for this was that for the first baby the mother doesn’t know what to do, so will therefore listen more to elders, in particular her mother. However, in subsequent pregnancies she will have more confidence, knowledge and experience so “goes her own way” moving towards modern medicine although may continue to use some traditional medicine.

*“These days we trust modern medicines more than “Khmer” traditional medicine. We still use a little bit to keep our traditional habit.”* [Mother]

There was a harmonious balance in families as well as individuals between the use of traditional and modern medicine. Grandparents were asked whether they minded if their daughters did not use traditional medicine during pregnancy or postpartum. There was a sense of tolerance for their daughters using modern medicine.

*“Yes, traditional medicine. I remember a lot in the past. Nowadays, society is changing so much and so quick, therefore, traditional medicines have been lost and I have forgotten. Our society nowadays, when woman is in labour pain, health centres are widely used. So, we don’t depend upon on those kinds of traditional medicines.”* [Grandfather]

### Neonatal Illness

The mothers who were interviewed could describe signs of a healthy or unwell baby.

*“When the baby is healthy they can suck very well, they can sleep a lot and their skin colour looks nice and fresh. But if they are not well they will not suck well, they cannot sleep, cry a lot and their body is thinner and pale”* [Mother]

One exception was the recognition of jaundice. All of the mothers whose baby had been admitted to hospital for the treatment of jaundice said they were surprised about the diagnosis and had not heard of it in a baby before.

*“Like my baby, when she was born and her skin was yellow, we thought that she is pretty and look lovely. But I don’t know it was an illness”* [Mother]

The main barrier to seeking healthcare was the cost of transportation, a one-way journey from rural areas of the health district to the referral hospital can cost up to $60. One respondent recalled the case of a baby who died in his village because the parents couldn’t find the money for transport

*“That’s right. If cannot borrow from anyone then would sell something. If we have cow, we sell cow yes. And some of them do not have money and cannot take their babies to hospital, and then die”* [Father]

Most interviewees said they would take a sick baby straight to a hospital or health centre. Although the decision to take a baby for medical treatment was described as being a family decision women felt empowered to be able to make the decision themselves even if other family members disagreed.

*“Is that transferred immediately after you were told to or you have to wait to discuss with your family member first?*

*“No transfer immediately”* [Mother]

## Discussion

Among those interviewed in this study, there was a stated move away from harmful practices that were conducted in the past such as food restriction, discarding colostrum, putting mud on the umbilical cord. The lack of food restriction is different from the practice described in neighbouring Laos where food restriction occurs early in pregnancy [12]. Most mothers said that they had had antenatal care, took iron supplements during pregnancy and had a facility based birth. The older generation seemed very accepting of this change. This differs from similar studies conducted in Ghana and Indonesia where grandmothers made healthcare decisions and enforced traditional practices [7, 13]. The reasons for this are likely to be complex. However, Cambodia’s traumatic history probably plays an important part. One third of Cambodia’s population was killed during the Khmer Rouge era, it is likely that knowledge of traditional medicine and practices was lost during this period potentially leaving a vacuum that modern knowledge and recommended practices have filled. For healthcare planners this is vital. This knowledge can be used to build relevant programmes, channelling scarce resources to teaching what is needed as opposed to imparting messages that are already known. For example in this population promoting early essential newborn care in healthcare facilities where mothers give birth [14].

The signs of a sick neonate were universally known, with the exception of recognising jaundice. Globally neonatal Jaundice is an important cause of neonatal morbidity and mortality. It is a common reason for hospital admission in the first week of life worldwide and one of the most common reasons for neurodevelopmental impairment in developing countries [15–17]. Community based interventions for the recognition and treatment of neonatal jaundice has the potential to be beneficial in this population.

There was no sense that medical help should not be sought, even from the poorest of the participants. Parents categorically stated that they would seek medical help even if opposed by the older generation. Proportion of blame to the mother for their child’s illness did not occur. Unlike that describe in a study conducted in Ghana which identified a significant influence by community members on health seeking behaviour and a frequent theme of blaming mothers for their infant’s illness [7]. The majority of participants stated that sick neonates must be taken to healthcare facilities for treatment and not treated at home or by traditional healers. The cost of transportation was stated as a major barrier and that delays in taking the neonate to healthcare facility were due to families trying to raise money to make the journey. A study conducted in Uganda examined the reasons that newborn infants there die, to do this they used a modified three delays model. This was comprised of delays in problem recognition or in deciding to seek care, delays in receiving care in a healthcare facility and delays in transportation. The authors concluded that household and healthcare facility related delays were the major contributors to neonatal mortality [18]. Again this is a very relative point for neonatal healthcare policy makers. The focus of such programmes in this area should be on transportation systems rather than teaching parents to recognise sick neonates.

Throughout the interviews and FGD it was apparent that grandmothers were involved in the care of their daughters and daughters in law during pregnancy, child birth and postpartum. However, there was no sense of dominance. Indeed, mothers seemed able to decide themselves whether to take traditional medicine or to partake of traditional practices, although some mothers, particularly for their first child, took and followed advice from the older generation.

The approach taken in choosing participants who had children admitted to hospital at the time of the study does have limitations. Drawing general conclusions from this group could potentially be problematic as this a group who have actively sort modern medicine. However no difference was apparent in beliefs and practices between groups participating in the study at the hospital and those participating in rural areas.

## Conclusions

In the population examined, traditional practices in late pregnancy and the post-partum period were no longer commonly performed. However, jaundice, a potentially serious neonatal condition, was not recognised. In this community neonatal interventions tailored to the recognition and treatment of neonatal jaundice have the potential to reduce neonatal morbidity and mortality.

## Additional files

Additional file 1: Focus group discussion topic guide. (PDF 89 kb)

Additional file 2: Semi structured interview topic guide. (PDF 89 kb)

## Abbreviations

AHC: Angkor Hospital for Children
FGD: Focus group discussions
PI: Principal investigator
SSI: Semi structured interviews

## References

[1] WHO. Accountability for maternal, newborn and child survival: the 2013 Update. WHO press; 2013.

[2] Progress Towards Millennium Development Goal 4: key facts and figures. September, 2013 [http://www.childinfo.org/mortality.html]

[3] Levels and trends in child mortality. Estimates developed by the UN Inter-agency Group for Child Mortality Estimation. Report 2013

[4] Rosato M, Laverack G, Grabman LH, Tripathy P, Nair N, Mwansambo C, Azad K, Morrison J, Bhutta Z, Perry H (2008). Community participation: lessons for maternal, newborn, and child health. Lancet.

[5] Lawn JE, Cousens S, Zupan J (2005). 4 million neonatal deaths: when? Where? Why?. Lancet.

[6] Turner C, Carrara V, Aye Mya Thein N, Chit Mo Mo Win N, Turner P, Bancone G, White NJ, McGready R, Nosten F (2013). Neonatal intensive care in a Karen refugee camp: a 4 year descriptive study. PLoS One.

[7] Engmann C, Adongo P, Aborigo RA, Gupta M, Logonia G, Affah G, Waiswa P, Hodgson A, Moyer CA (2013). Infant illness spanning the antenatal to early neonatal continuum in rural northern Ghana: local perceptions, beliefs and practices. J Perinatol.

[8] Herlihy JM, Shaikh A, Mazimba A, Gagne N, Grogan C, Mpamba C, Sooli B, Simamvwa G, Mabeta C, Shankoti P (2013). Local perceptions, cultural beliefs and practices that shape umbilical cord care: a qualitative study in southern province, zambia. PLoS One.

[9] MOH (2010). Cambodia demographic and health survey 2012.

[10] Herbert HK, Lee AC, Chandran A, Rudan I, Baqui AH (2012). Care seeking for neonatal illness in low- and middle-income countries: a systematic review. PLoS Med.

[11] Angkor Hospital for Children [http://angkorhospital.org/]

[12] Alvesson HM, Lindelow M, Khanthaphat B, Laflamme L (2013). Changes in pregnancy and childbirth practices in remote areas in Lao PDR within two generations of women: implications for maternity services. Reprod Health Matters.

[13] Sutan R, Berkat S (2014). Does cultural practice affects neonatal survival- a case control study among low birth weight babies in Aceh Province, Indonesia. BMC Pregnancy Childbirth.

[14] WHO (2014). Action plan for healthy newborn infants in the western pacific region (2014–2020).

[15] Olusanya BO, Akande AA, Emokpae A, Olowe SA (2009). Infants with severe neonatal jaundice in Lagos, Nigeria: incidence, correlates and hearing screening outcomes. Tropical Med Int Health.

[16] Slusher TM, Zipursky A, Bhutani VK. A global need for affordable neonatal jaundice technologies. Semin Perinatol. 2011;35(3):185–91. 10.1053/j.semperi.2011.02.014 21641493

[17] Bhutta ZA, Darmstadt GL, Hasan BS, Haws RA (2005). Community-based interventions for improving perinatal and neonatal health outcomes in developing countries: a review of the evidence. Pediatrics.

[18] Waiswa P, Kallander K, Peterson S, Tomson G, Pariyo GW (2010). Using the three delays model to understand why newborn babies die in eastern Uganda. Tropical Med Int Health.

