# Supplementary material for: Beliefs and practices during pregnancy, post-partum and in the first days of an infant’s life in rural Cambodia
Source: BMC Pregnancy Childbirth. 2017 Apr 12;17:116. doi: 10.1186/s12884-017-1305-9 (PMC5389162; doi:10.1186/s12884-017-1305-9)
Supplement: Supplementary file 2 — Semi structured interview topic guide. (PDF 89 kb) [file 12884_2017_1305_MOESM2_ESM.pdf]

Health perception, beliefs and practices at the time of delivery and in the neonatal period in rural Cambodia

Semi Structured Interview Topic Guide

Study code: HPB -|__|__|__| **Facilitator Initials:** |__|__|__| **Note-taker Initials:** |__|__|__|

Date: |__|__|/|__|__|__|__| /|__|__| Audio file: SSI - |__|__|__| __|__|__|- |__|__|__|

Has Consent Been Signed?

Introduction

⇒ General purpose of the study
⇒ Aims of the interview and expected duration
⇒ Who is involved in the process (other participants)
⇒ Why the participant's cooperation is important
⇒ Check position of the tape recorder
⇒ What will happen with the collected information and how the participant/target group will benefit
⇒ Any questions?

Complete the CRF

Domain	Topic and Probes
**Delivery**	1. Do you know of any problems that can happen to a woman during delivery?<br>2. Can any of these problems affect the baby?<br>3. Why do you think some women and babies get problems during delivery?<br>4. Some babies are born and do not breath. Why do you think this happens?<br>5. What is the role of the father during delivery?<br>6. Do any other family members have important roles during delivery?<br>7. Why does a baby have an umbilical cord?<br>8. When a baby is born at home what is used to cut the umbilical cord?<br>**Prompts:**<br>Would you be worried if the mother in labour and childbirth had:<br>Fever<br>Waters broken but no labour pains<br>Bleeding from the vagina before delivery<br>Bleeding from the vagina after delivery<br>Had more than one day of labour pains

Domain	Topic and Probes
**Newborn**	1. Are there any special things that must be done after a baby is born and who does them?<br>2. When does a child get their name?<br>3. Who chooses the child's name?
**Neonatal illness**	1. How do you know a newborn baby is healthy?<br>2. When should breast feeding start and why?<br>3. Are there any signs that a baby is ill? (each answer will be examined by asking why each happens)<br>4. Who can tell if a baby is sick?<br>Prompts:<br>Would you be worried about a baby who had:<br>Fever<br>Breathing fast<br>Poor sucking<br>Yellow skin<br>Abnormal movements
**Health care seeking**	1. Whose responsibility is it to decide whether to take a sick baby for help?<br>2. How is the decision made to take a baby for help?<br>3. Who pays for treatment?<br>4. When would a mother seek help for her baby?<br>5. Where would they go first?<br>6. How would they get there?<br>7. Some babies die in the first one month of life can you explain why?
**Ethics**	1. Should all sick babies be referred to hospital?<br>2. Who should decide whether a sick baby gets sent to hospital?<br>3. What should happen if a parent refuses to take a baby to hospital?<br>4. What do you understand by the term “human rights”?<br>5. Does a baby have the same “human rights” as an older child/adult?<br>6. When does life begin?

Closing

⇒ Is there anything else you think is important in diagnosing malaria that we have not talked about?
⇒ Summarise
⇒ Thank the participant
⇒ Provide extra information and contacts to participants
